# Course of untreated depression in people with tuberculosis in Ethiopia: a cohort study

**DOI:** 10.1192/j.eurpsy.2023.231

**Published:** 2023-07-19

**Authors:** F. A. Getahun

**Affiliations:** School of Public Health, Bahir Dar University, Bahir Dar, Ethiopia

## Abstract

**Introduction:**

Co-morbid depression is common in people with tuberculosis (TB) but little is known about the course over time.

**Objectives:**

Our objectives were to determine the level of remission and factors associated with failure to remit in depressive symptom scores in people with TB undergoing treatment in primary care facilities in Ethiopia.

**Methods:**

We assessed 648 people with newly diagnosed TB for depressive symptoms using Patient Health Questionnaire (PHQ-9) at the time of starting anti-TB medication, and again at two and six months. Remission was defined as more than 50% reduction in baseline depressive symptom scores. We analyzed factors associated with failure of depressive symptom score to remit at the end of the follow up using multilevel mixed-effects logistic regression by taking individuals as nested within 14 health institutions. Adjustment was made for socio-demographic characteristics, baseline depression score, stigma, type of TB, outcome of TB treatment, perceived severity of TB, substance use, perceived social support, substance use, and HIV status.

**Results:**

Compared to the baseline, the mean PHQ-9 scores declined at two months (Hedge’s G: 0.82; 95%CI: 0.71, 0.94) and six months (Hedge’s G: 1.20; 95%CI: 1.08, 1.33). However, depressive symptom scores failed to remit in 176 (33.1%) of the 532 people with TB who completed the follow up. . Stigma (AOR: 2.23; 95%CI: 1.09, 4.55), older age (AOR: 2.2; 95%CI: 1.13, 4.29), and treatment completion without a bacteriological proof of cure (treatment complete as compared to treatment cure) (AOR: 2.47; 95%CI: 1.37, 4.48) were independent predictors of failure of depressive symptom score to remit. Surprisingly, baseline lower depressive symptom score was more persistent than higher baseline depressive symptom score (AOR: 2.93; 95%CI: 1.56, 5.47).

**Conclusions:**

In one-third of people with newly diagnosed TB, baseline depressive symptom scores did not remit after full course of TB treatment. TB treatment guidelines require in-built mental health component. Studies are required to understand course of depression beyond six months and effective interventions in this population.

**Disclosure of Interest:**

None Declared

